# Esophageal Eosinophilia in Pediatric and Young Adult Patients with Inflammatory Bowel Disease: Diagnosis, Clinical Course, and Treatment Outcomes

**DOI:** 10.3390/children13070889

**Published:** 2026-07-02

**Authors:** Jarrett Rardon, Elizabeth Erwin, Brendan Boyle, Hilary K. Michel, John M. Russo, Raul E. Sanchez, Rajitha D. Venkatesh, Ross M. Maltz

**Affiliations:** 1 Division of Pediatric Gastroenterology, Hepatology, and Nutrition, Nationwide Children’s Hospital, Columbus, OH 43205, USA; jarrett.rardon@nationwidechildrens.org (J.R.); brendan.boyle@nationwidechildrens.org (B.B.); hilary.michel@nationwidechildrens.org (H.K.M.); john.russo@nationwidechildrens.org (J.M.R.); raul.sanchez@nationwidechildrens.org (R.E.S.); rajitha.venkatesh@nationwidechildrens.org (R.D.V.); 2 Department of Pediatrics, The Ohio State University Wexner Medical Center, Columbus, OH 43210, USA; elizabeth.erwin@nationwidechildrens.org; 3 Division of Allergy and Immunology, Nationwide Children’s Hospital, Columbus, OH 43205, USA

**Keywords:** Crohn’s disease, ulcerative colitis, eosinophilic esophagitis

## Abstract

**Highlights:**

**What are the main findings?**

- Esophageal eosinophilia is frequently identified in patients with inflammatory bowel disease during routine endoscopic evaluation of disease activity, often in the absence of symptoms of esophageal dysfunction.
- Histologic remission rates of esophageal eosinophilia were similar between treated and untreated patients; remission was observed only among patients receiving low-dose proton pump inhibitor therapy.

**What are the implications of the main findings?**

- Esophageal eosinophilia identified in patients with inflammatory bowel disease may represent a distinct entity and does not necessarily indicate eosinophilic esophagitis.
- In asymptomatic patients, a strategy of clinical monitoring for the development of esophageal dysfunction symptoms, with consideration of repeat esophagogastroduodenoscopy, may be appropriate.

**Abstract:**

**Background/Objectives**: This study evaluates the clinical presentation and treatment outcomes of esophageal eosinophilia (EE) in patients with inflammatory bowel disease (IBD). It compares outcomes based on the timing of diagnosis (simultaneous vs. EE after IBD diagnosis) and treatment (treated vs. untreated EE). **Methods**: This retrospective cohort study included patients ≤ 22 years with IBD and EE (≥15 eosinophils/high-powered field {eos/HPF}) between July 2017 and December 2023. Clinical, endoscopic, histologic, and EE treatment data were collected. EE histologic remission defined as <15 eos/HPF was assessed on repeat esophagogastroduodenoscopy (EGD). Groups were compared using standard statistical tests, with *p* < 0.05 considered significant. **Results**: Of fifty-six patients with IBD and EE, thirteen (23%) were diagnosed with EE and IBD simultaneously and forty-three (77%) patients were diagnosed with EE after IBD. Most were diagnosed with EE incidentally. Esophageal symptoms and histologic findings between patients diagnosed simultaneously vs. EE after IBD were similar. Among patients treated for EE (n = 28) and those who were untreated (n = 28), there were significant differences in esophageal symptoms (54% vs. 0%; *p* < 0.01) and median esophageal eosinophil count (30 vs. 22 eos/HPF; *p* = 0.003). However, among patients with a repeat EGD, EE histologic remission rates were similar between those who were treated vs. untreated (35% vs. 47%; *p* = 0.5). **Conclusions**: We found that for patients with both IBD and EE, most were diagnosed incidentally with EE after IBD diagnosis. The observation of spontaneous histologic esophageal remission in a subset of patients suggests that incidental EE in patients with IBD may not always represent eosinophilic esophagitis (EoE) and should not automatically lead to a lifelong diagnosis.

## 1. Introduction

Inflammatory bowel disease (IBD) and eosinophilic esophagitis (EoE) are chronic gastrointestinal conditions that occur due to overactivation of the mucosal immune system in genetically susceptible patients [1,2]. Patients with IBD may present with or subsequently develop esophageal eosinophilia (EE), defined as having ≥15 eosinophils/high-power field (eos/HPF). EoE is diagnosed by combining clinical symptoms of esophageal dysfunction with an upper endoscopy demonstrating esophageal eosinophilia (≥15 eos/HPF) in tissue biopsies after excluding other causes such as Crohn’s disease (CD) of the esophagus or reflux esophagitis [1]. There is limited pediatric literature differentiating between these entities among children with IBD [3].

Studies describing the clinical course of pediatric patients with EoE and IBD are limited and heterogenous. In these studies, the estimated prevalence of pediatric patients with co-occurring EoE and IBD varies widely from 0.35 to 8.8% [3,4,5]. The prevalence reported in adults with IBD and co-occurring EoE was 0.10% and 0.0078% [6,7]. Risk factors associated with developing EoE among children with IBD include having CD as opposed to ulcerative colitis (UC) and having a history of food allergies [3,5]. In the largest study describing IBD and EoE in 67 children, treatment with an anti-tumor necrosis factor alpha (anti-TNFα) was found to have a protective effect, preventing the development of EoE [3]. The timing of diagnosis of EoE relative to the timing of IBD diagnosis was described in two studies which found that the majority of patients developed EoE after being diagnosed with IBD [5,8]. Similarly, only one pediatric study included the presence of symptoms of esophageal dysfunction in their definition of EoE. This raises the question as to whether they truly have EoE or rather EE. Only one study evaluated treatment outcomes [8]. They found that the frequency of esophageal histologic remission in patients with EoE and IBD was similar to that found in those with EoE only, though the study was limited to only 29 patients with EoE and IBD and did not consider symptoms of esophageal dysfunction in their diagnostic criteria [8].

Given the unanswered questions that remain and the common occurrence of EE in patients with IBD, more information about diagnosis and treatment is needed. The primary objectives of this study were to (1) evaluate whether presentations of EE and outcomes from repeat esophagogastroduodenoscopy (EGD) were different in patients diagnosed with IBD and EE simultaneously compared to patients diagnosed with EE after IBD diagnosis; (2) evaluate whether presentations of EE and outcomes from repeat EGD were different in patients treated for EE vs. those who were untreated for EE.

## 2. Materials and Methods

### 2.1. Study Design

This is a single-center retrospective cohort study of pediatric and young adult patients aged ≤22 years diagnosed with IBD between 1 July 2017 and 12 December 2023, who were found to have EE on EGD. A diagnosis of EE was based upon histologic criteria of ≥15 eos/HPF in either the proximal or distal esophageal biopsies. Patients were included if they were diagnosed with EE and IBD simultaneously and if they were diagnosed with EE after IBD. Patients diagnosed with EE prior to the diagnosis of IBD were excluded. Patients were identified via cross-referencing our internal EoE and IBD databases at a tertiary academic medical center. Our internal EoE database tracks any patient with ≥15 eos/HPF found on esophageal histology.

Clinical data was collected, including age, past medical history of allergic and atopic disease (i.e., asthma, eczema, IgE-mediated food allergies), family history of IBD and EoE, and IBD type (CD, UC, or IBD-unclassified {IBD-U}). IBD activity at the time of EE diagnosis was assessed using the recorded physician global assessment (PGA). The PGA is a validated tool for assessing disease activity in IBD [9,10]. By integrating patient symptoms, physical examination findings, laboratory parameters, and functional status, it provides a comprehensive evaluation of overall disease severity. EE diagnosis date, indication for EGD, and presence of symptoms of esophageal dysfunction (dysphagia, epigastric pain, vomiting, and food impaction) around the time of EGD as documented in the electronic medical record (EMR) were recorded. Endoscopic data included the physician-documented macroscopic esophageal findings at the time of EGD. Endoscopic appearance was categorized as EoE when edema, rings, exudates, or furrows were present; reflux-associated eosinophilia when distal erythema or distal erosions were identified in the absence of rings or furrows; esophageal CD when aphthous ulcers or patchy inflammation were observed; or normal when no macroscopic abnormalities were noted. Histological data included peak esophageal eosinophil count, presence of basal cell hyperplasia (BCH) and/or presence of lamina propria fibrosis (LPF). Treatments for both IBD and EE were recorded and treatment choices were based on the physician’s discretion. High-dose proton pump inhibitor (PPI) was considered 1 mg/kg twice a day (max dose 40 mg twice daily). All dosages less than 1 mg/kg were considered to constitute low-dose PPI use. The results of repeat EGD, including esophageal endoscopic and histologic findings, were recorded. Histological remission was defined as having less than 15 eos/HPF on repeat EGD.

Study variables were summarized using descriptive statistics including medians and interquartile ranges (IQRs). Comparisons between patients who have been simultaneously diagnosed with EE and IBD and patients with EE after IBD were performed using chi-square or Fisher’s exact test for categorical data and the Mann–Whitney test for continuous data. In addition, a similar analysis was also completed which compared patients treated for EE and patients who did not receive treatment for EE. All data were analyzed using the statistical software GraphPad Prism version 10.6 (GraphPad Software, San Diego, CA, USA) with two-sided *p*-values < 0.05 considered statistically significant.

### 2.2. Ethical Considerations

Institutional review board approval (Study00003788) was obtained.

## 3. Results

### 3.1. Demographics

In total, 56 patients were found to have IBD and EE and were included in the study (Table 1). Of these, 75% (42/56) were male, 84% (47/56) were white, and 77% (43/56) had CD. The median age at IBD diagnosis was 12 years (IQR 8.7–15.3) and the median age at EE diagnosis was 14.2 years (IQR 11–17.2). Family history of IBD was common (21%), while family history of EoE occurred in fewer than 1% of patients. Personal history of atopic co-morbidities such as eczema, IgE-mediated food allergies, or asthma occurred in 40%, 34%, and 27% of patients.

Thirteen (23%) patients were diagnosed with EE and IBD simultaneously and forty-three (77%) patients were found to have EE after IBD diagnosis. The demographic data between the two groups were similar; however, a greater proportion of patients who were diagnosed with EE after IBD were more likely to be diagnosed with CD (vs. UC) than in the simultaneous group (*p* = 0.02) (Table 1). Among the patients diagnosed with EE after IBD at or near the time of EE diagnosis, their IBD PGA was classified as quiescent 77% (n = 33), mild 21% (n = 9), and moderate 2% (n = 1). The IBD therapies patients were receiving at the time of EE diagnosis included anti-TNFα 67% (n = 29), mesalamines 12% (n = 5), immunomodulator 9% (n = 4), ustekinumab 7% (n = 3) and steroids 5% (n = 2).

Of the patients diagnosed with EE, half were treated (n = 28) and half were untreated (n = 28). Demographic data, sex, age, allergic and atopic disease history, and IBD diagnosis type were similar between the treated and untreated groups (Table 2).

### 3.2. Esophageal Symptoms at Diagnosis and Endoscopic and Histologic Findings

In the total population, only 5% of patients (3/56) had “evaluation for EoE” as a listed indication for their EGD based on presenting with symptoms of esophageal dysfunction, while 27% (15/56) had documented esophageal dysfunction before and after the EGD (Table 1). Thirty-eight percent (21/56) of patients had macroscopic findings on the EGD to suggest EoE was the diagnosis and 54% (30/56) had a normal-appearing esophagus (Table 1). The median peak eosinophil count was 29 eos/HPF (IQR 20–29), with 68% of patients having BCH and 20% having LPF.

The symptoms of esophageal dysfunction and histologic findings were similar between patients diagnosed with EE and IBD simultaneously and patients diagnosed with EE after IBD (Table 1). Among patients diagnosed with EE after IBD, 72% had an EGD to assess for IBD endoscopic healing and 23% had an EGD to assess for active IBD.

Symptoms of esophageal dysfunction, macroscopic esophageal findings, and median eos/HPF differed among the patients treated for EE compared to those who did not receive treatment for EE (Table 2). Treated patients were more likely to report esophageal symptoms (54% vs. 0%; *p* < 0.01), have macroscopic esophageal findings consistent with EoE (57% vs. 18%; *p* = 0.04), and have a higher median peak esophageal eosinophil count (30 eos/HPF (IQR 28–42) vs. 22 eos/HPF (IQR 19–30); *p* = 0.003) compared to patients who remained untreated for EE. The percentages of patients with BCH and LPF were similar between the groups.

### 3.3. Treatment for Esophageal Eosinophilia and Clinical Outcomes

In the total population, 64% (36/56) of patients had a repeat EGD (Table 3). No treatment for EE occurred in 50% of patients, whereas 28% of patients started on low-dose PPI, 16% started high-dose PPI, 4% started topical steroids, and 2% started dietary elimination therapy. On repeat EGD, 53% of patients had a normal-appearing esophagus and 42% were considered in histologic remission for EE.

There were no statistically significant differences in treatments for EE, EE histologic remission rates, median peak esophageal eosinophil counts, or the presence of BCH and LPF between patients diagnosed with EE and IBD simultaneously and patients diagnosed with EE after IBD. Patients who were diagnosed with EE and IBD simultaneously had a higher rate of repeat EGD compared to patients who were diagnosed with EE after IBD. There were also similar rates of EE histologic remission among patients with a repeat EGD with CD (43%, n = 12) compared to patients with UC/IBD-U (38%, n = 3) (*p* > 0.99).

At the time of repeat EGD, patients continued the prescribed treatment for EE. The IBD therapies patients were receiving at the time of repeat EGD included anti-TNFα 63% (n = 15), mesalamines 21% (n = 5), ustekinumab 8% (n = 2), enteral therapy 4% (n = 1), and no medications 4% (n = 1). Five patients changed IBD treatments over the study period. A similar percentage of patients had a repeat EGD to assess EE in the group of patients treated for EE compared to those who went untreated (61% vs. 68%, *p* = 0.78) (Table 4). Rates of EE histologic remission (35% vs. 47%; *p* = 0.5) and esophageal histologic findings (20 eos/HPF (IQR 0–30) vs. 15 eos/HPF (IQR 0–43.5); *p* = 0.6) were not statistically different between treated and untreated groups. The six patients with histologic remission in the treated for EE group were all treated with low-dose PPI.

## 4. Discussion

Our study suggests that patients with EE and IBD should not automatically receive a lifelong diagnosis of EoE because some patients may have EE due to CD of the esophagus or reflux esophagitis. In addition, a subset of patients had spontaneous esophageal histologic remission. Only 27% of patients had documented symptoms of esophageal dysfunction around the time of their EGD. Reassessment for IBD endoscopic mucosal healing was the most common indication for EGD at the time of EE diagnosis; this finding was similar to those from prior studies [3,4]. Macroscopic esophageal changes were found in <40% of patients, despite the eosinophil count being ≥15 eos/HPF. Only 50% of patients were treated for EE in our cohort, with variable treatment regimens including high- or low-dose PPI, topical steroids, or diet therapy. Among treated patients with esophageal remission on repeat EGD, each received low-dose PPI, while nearly half of the patients who were untreated were in remission too. This further emphasizes that not all patients with EE and IBD also have EoE and assessing symptoms of esophageal dysfunction is important.

Three studies have evaluated the frequency of EoE and IBD diagnoses at different timepoints and the relevance of this timing is not understood [5,8,11]. In a sample of 29 patients with EoE and IBD, Hudson et al. (2025) found a similar rate of patients diagnosed with EoE and IBD simultaneously (21%) compared to rates of EoE diagnoses made after IBD (76%) [8]. Splawski et al. (2022) found a higher percentage of patients with UC were diagnosed with EoE simultaneously and a higher percentage of patients with CD were subsequentially diagnosed with EoE, which was similar to our study [5]. Our study is the only study that evaluated treatment outcomes among these two groups and found no difference in esophageal histologic remission rates.

Treatment decisions were made according to physicians’ discretion. Treated patients were more likely to report symptoms of esophageal dysfunction, have macroscopic esophageal findings consistent with EoE, and have a higher peak esophageal eosinophil count compared to the untreated patients. EE histologic remission rates were similar between the treated and untreated groups. On repeat EGD, 47% of patients who did not receive treatment for EE had spontaneous histologic remission of EE and 36% of patients treated for EE were in histologic remission. In addition, only the patients who were prescribed low-dose PPI treatment were in histologic remission. Spontaneous remission in patients with EE who were asymptomatic or minimally symptomatic was frequently observed, as has been previously described [12]. These observations suggest a potential for spontaneous remission in EE and that the apparent benefit seen with low-dose PPI, a nonstandard regimen for EoE, raises important questions about optimal management for patients with IBD and EE.

Previous studies are mixed as to whether the anti-TNF agents increase one’s risk of developing EoE as opposed to acting as a protective against developing EoE among pediatric patients with IBD [3,5,13]. The majority of patients in our study were on an anti-TNF agent at the time of EE diagnosis. Further research is needed to clarify associations between anti-TNF exposure and development of EE.

EoE guidelines require esophageal eosinophilia of ≥15 eos/HPF in the setting of symptoms of esophageal dysfunction for a diagnosis of EoE [1]. However, the majority of prior studies among IBD patients used only the histologic criteria, which may overestimate rates of EoE by inadvertently including patients with reflux esophagitis or CD of the esophagus without true symptoms of esophageal dysfunction [4,5,8,13]. In addition, epigastric abdominal pain and vomiting are considered to be symptoms of esophageal dysfunction. They are also symptoms that can be seen in patients with IBD and may not indicate esophageal dysfunction. In the future, the diagnosis of EoE could be more specific if it includes the Eosinophilic Esophagitis Histologic Scoring System (EoE-HSS), which considers other histologic findings [14]. Given its chronic nature, requiring lifelong treatment, clinicians should consider only diagnosing EoE if they have identified esophageal eosinophil counts ≥ 15 eos/HPF and true symptoms of esophageal dysfunction.

The limitations of our study include the discrepancy between the number of patients with symptoms of esophageal dysfunction and the proportion of patients who had “evaluation for EoE” listed as an indication for endoscopy. The discrepancy is likely because, prior to EGD, not all patients reported or were assessed for symptoms of esophageal dysfunction, and most were only assessed after receiving a diagnosis of EE. The retrospective nature of the study potentially limited the number of patients reporting symptoms of esophageal dysfunction. In addition, only 64% of patients underwent repeat EGD, which could have created a selection bias among patients who underwent a follow-up EGD. The absence of statistically significant differences may reflect limited statistical power rather than true equivalence between groups. Further research should include a prospective study among patients with IBD assessing symptoms of esophageal dysfunction at the time of EGD using validated tools. In addition, while we reported remission rates stratified by treatment approaches, we were unable to assess treatment adherence in this retrospective study.

## 5. Conclusions

In summary, most patients with IBD and EE were incidentally diagnosed with EE after IBD during routine IBD endoscopic reassessment and had no macroscopic findings of EoE. Nearly 50% of patients had spontaneous remission of EE without treatment and some patients responded to low-dose PPI. Taken together, these findings suggest that if a patient is diagnosed with IBD and EE, but the diagnosis of EoE is not certain due to a lack of esophageal symptoms or macroscopic findings of EoE, it may be appropriate to monitor a patient off EoE specific therapy with routine symptom reassessment and repeat EGD or transnasal esophagoscopy, prior to diagnosing them with EoE, a chronic disorder which requires lifelong therapy.

## Figures and Tables

**Table 1:** Demographics.

	Total N = 56	EE and IBD Simultaneously N = 13	EE After IBD N = 43	*p*-Value
**Sex n (%)**				1.0
Male	42 (75%)	10 (77%)	32 (74%)	
**Race n (%)**				0.42
White	47 (84%)	10 (77%)	37 (86%)	
Non-White	9 (16%)	3 (23%)	6 (14%)	
**Age Median (IQR)**				
Median age at IBD diagnosis	12 (IQR 8.7–15.3)	15.3 (IQR 13–17.4)	11.2 (IQR 8.3–14.3)	0.15
Median age at EE diagnosis	14.2 (IQR 11–17.2)	15.3 (IQR 13–17.4)	13.6 (IQR 11–17)	0.59
**Medical History n (%)**				
Eczema	22 (40%)	6 (46%)	16 (37%)	0.75
Food Allergies	19 (34%)	5 (38%)	14 (33%)	0.75
Asthma	15 (27%)	5 (38%)	10 (23%)	0.3
**Family History n (%)**				
EoE	1 (<1%)	0 (0%)	1 (2%)	1.0
IBD	12 (21%)	4 (31%)	8 (19%)	0.44
**IBD Diagnosis n (%)**				**0.02**
Crohn’s disease	43 (77%)	8 (62%)	35 (81%)	
Ulcerative colitis/IBD-U	13 (23%)	5 (28%)	8 (19%)	
**EGD indication n (%)**				**<0.01**
IBD Repeat	31 (55%)	0 (0%)	31 (72%)	
Active IBD	22 (40%)	13 (100%)	10 (23%)	
EoE Symptoms	3 (5%)	1 (8%)	2 (5%)	
**Symptoms of Esophageal Dysfunction n (%)**				1.0
Yes	15 (27%)	3 (24%)	12 (28%)	
No	51 (73%)	10 (76%)	31 (72%)	
**Esophageal Findings n (%)**				0.40
CD	3 (5%)	1 (8%)	2 (5%)	
EoE	21 (38%)	7 (54%)	14 (33%)	
Reflux	2 (4%)	0 (0%)	2 (5%)	
Normal	30 (54%)	5 (38%)	25 (57%)	
**Esophageal Histology n (%)**				
Median Peak eos/HPF (IQR)	29 (20–35.5)	25 (20–30)	30 (20–37.5)	0.96
BCH	38 (68%)	10 (77%)	28 (65%)	0.51
LPF	11 (20%)	3 (23%)	8 (19%)	0.70

EE—esophageal eosinophilia; IBD—inflammatory bowel disease; n—number; IQR—interquartile range; EoE—eosinophilic esophagitis; IBD-U—inflammatory bowel disease-unclassified; EGD—esophagogastroduodenoscopy; CD—Crohn’s disease; eos/HPF—eosinophils/high-powered field; BCH—basal cell hyperplasia; LPF—lamina propria fibrosis.

**Table 2:** Demographics based on treatment status.

	Total N = 56	Treated N = 28	Untreated N = 28	
**Sex n (%)**				
Male	42 (75%)	19 (67%)	23 (82%)	0.36
**Race n (%)**				0.43
White	47 (84%)	23 (82%)	24 (85%)	
Non-White	9 (16%)	5 (18%)	4 (15%)	
**Age Median (IQR)**				
Median age at IBD diagnosis	12 (IQR 8.7–15.3)	12 (IQR 10–15)	10 (IQR 8–14)	0.29
Median age at EE diagnosis	14.2 (IQR 11–17.2)	15 (IQR 12–17)	13 (IQR 10–17)	0.27
**Medical History n (%)**				
Eczema	22 (40%)	12 (43%)	10 (36%)	0.78
Food Allergies	19 (34%)	9 (32%)	10 (36%)	1.0
Asthma	15 (27%)	7 (25%)	8 (28%)	1.0
**Family History n (%)**				
EoE	1 (<1%)	1 (3%)	0	1.0
IBD	12 (21%)	9 (32%)	3 (11%)	0.1
**IBD Diagnosis n (%)**				0.2
Crohn’s disease	43 (77%)	19 (68%)	24 (86%)	
Ulcerative colitis/IBD-U	13 (23%)	9 (32%)	4 (14%)	
**EGD indication n (%)**				0.26
IBD Repeat	31 (55%)	14 (50%)	17 (61%)	
Active IBD	22 (40%)	11 (39%)	11 (39%)	
EoE Symptoms	3 (5%)	3 (11%)	0 (0%)	
**Symptoms of Esophageal Dysfunction n (%)**				**<0.01**
Yes	15 (27%)	15 (54%)	0 (0%)	
No	41 (73%)	13 (46%)	28 (100%)	
**Esophageal Findings n (%)**				**0.04**
CD	3 (5%)	2 (7%)	1 (4%)	
EoE	21 (38%)	16 (57%)	5 (18%)	
Reflux	2 (4%)	0 (0%)	2 (7%)	
Normal	30 (54%)	10 (36%)	20(71%)	
**Esophageal Histology n (%)**				
Median Peak eos/hpf (IQR)	29 (20–35.5)	30 (28–43)	22 (19–30)	**0.003**
BCH	38 (68%)	20 (71%)	18 (64%)	0.78
LPF	11 (20%)	8 (29%)	3 (11%)	0.18

n—number; IQR—interquartile range; IBD—inflammatory bowel disease; EE—esophageal eosinophilia; EoE—eosinophilic esophagitis; IBD-U—inflammatory bowel disease-unclassified; EGD—esophagogastroduodenoscopy; CD—Crohn’s disease; eos/HPF—eosinophils/high-powered field; BCH—basal cell hyperplasia; LPF—lamina propria fibrosis.

**Table 3:** Repeat esophagogastroduodenoscopy outcomes: timing of diagnosis.

	Total N = 56	EE and IBD Simultaneously N = 13	EE After IBD N = 43	*p*-Value
**Treatments**				0.3
Low-dose PPI	16 (28%)	3 (23%)	13 (30%)	
High-dose PPI	9 (16%)	1 (8%)	8 (19%)	
Topical Steroids	2 (4%)	1 (8%)	1 (2%)	
Diet therapy	1 (2%)	1 (8%)	0 (0%)	
No treatment	28 (50%)	7 (54%)	21 (49%)	
**Repeat EGD n (%)**	N = 36 (64%)	N = 12 (92%)	N = 24 (56%)	**0.02**
**Months to repeat EGD Median (IQR)**	10 (6–15)	11.5 (7.5–15)	8.5 (5–14.5)	0.55
**Esophageal Findings n (%)**				
CD	0 (0%)	0 (0%)	0 (0%)	0.27
EoE	16 (44%)	4 (33%)	12 (50%)	
Reflux	1 (3%)	1 (8%)	0 (0%)	
Normal	19 (53%)	7 (59%)	12 (50%)	
**Remission n (%) < 15 eos/hpf**	15 (42%)	4 (33%)	11 (46%)	0.72
**Esophageal Histology n (%)**				
Median Peak eos/HPF (IQR)	16.5 (0–43)	18 (8–50)	15 (0–33)	0.44
BCH n, %	17 (47%)	7 (58%)	10 (42%)	0.48
LPF n, %	6 (17%)	2 (17%)	4 (17%)	1.0

EE—esophageal eosinophilia; IBD—inflammatory bowel disease; PPI—proton pump inhibitor; EGD—esophagogastroduodenoscopy; n—number; CD—Crohn’s disease; EoE—eosinophilic esophagitis; eos/HPF—eosinophils/high-powered field; IQR—interquartile range; BCH—basal cell hyperplasia; LPF—lamina propria fibrosis.

**Table 4:** Repeat esophagogastroduodenoscopy outcomes: esophageal eosinophilia treatment.

	Total N = 56	Treated N = 28	Untreated N = 28	*p*-Value
**Repeat EGD n (%)**	N = 36 (64%)	N = 17 (61%)	N = 19 (68%)	0.78
**Months to Repeat EGD Median (IQR)**	10 (6–15)	8 (3–11)	13 (8–22)	**0.005**
**Esophageal Findings n (%)**				
CD	0 (0%)	0 (0%)	0 (0%)	0.62
EoE	16 (44%)	8 (47%)	8 (42%)	
Reflux	1 (3%)	1 (6%)	0 (0%)	
Normal	19 (53%)	8 (47%)	11 (58%)	
**Remission n (%) < 15 eos/hpf**	15 (42%)	6 (35%)	9 (47%)	0.5
**Esophageal Histology n (%)**				
Median Peak eos/HPF (IQR)	16.5 (0–43)	20 (0–30)	15 (0–43.5)	0.6
BCH n, (%)	17 (47%)	9 (53%)	8 (42%)	0.74
LPF n, (%)	6 (17%)	2 (12%)	4 (21%)	0.66

EGD—esophagogastroduodenoscopy; n—number; CD—Crohn’s disease; EoE—eosinophilic esophagitis; eos/HPF—eosinophils/high-powered field; IQR—interquartile range; BCH—basal cell hyperplasia; LPF—lamina propria fibrosis.

## Data Availability

The data presented in this study are available on request from the corresponding author due to (PHI).

## Abbreviations

The following abbreviations are used in this manuscript:

EE	Esophageal Eosinophilia.
IBD	Inflammatory Bowel Disease.
EoE	Eosinophilic Esophagitis.
Eos/HPF	Eosinophils per High-Power Field.
EGD	Esophagogastroduodenoscopy.
CD	Crohn’s Disease.
UC	Ulcerative colitis.
IBD-U	IBD-Unspecified.
Anti-TNFα	Anti-Tumor Necrosis Factor Alpha.
IQR	Interquartile Range.
PPI	Proton Pump Inhibitor.
BCH	Basal Cell Hyperplasia.
LPF	Lamin Propria Fibrosis.
